# Secular trends and sociodemographic determinants of thinness, overweight and obesity among Chinese children and adolescents aged 7–18 years from 2010 to 2018

**DOI:** 10.3389/fpubh.2023.1128552

**Published:** 2023-05-04

**Authors:** Chengyue Li, Mingxuan Zhang, Alimujiang Yimiti Tarken, Yuping Cao, Qian Li, Hao Wang

**Affiliations:** Institute of Physical Education, Xinjiang Normal University, Urumqi, China

**Keywords:** children and adolescents, overweight, obesity, thinness, sociodemographic determinants, secular trends

## Abstract

**Background:**

Most studies have focused on overweight/obesity and its secular trend, with insufficient studies on the factors influencing thinness and trends recently. To examine the trends of prevalence and sociodemographic determinants of thinness, overweight, and obesity among Chinese children and adolescents aged 7 to 18 years from 2010 to 2018.

**Methods:**

This study was based on cross-sectional data of 11,234 children and adolescents aged 7 to 18 years from the Chinese Family Panel Studies (CFPS) in 2010, 2014, and 2018, including anthropometric and sociodemographic characteristics variables. The nutritional status of each individual was determined according to China and WHO criteria. The demographic characteristics of different subgroups were tested by chi-square, and log-binomial regression was used to analyze the trend of prevalence and the relationship between sociodemographic characteristics and different nutritional statuses.

**Results:**

After adjusting for age, from 2010 to 2018, the overall prevalence of thinness decreased, and the prevalence of overweight increased in Chinese children and adolescents. The overall prevalence of obesity declined in boys and increased in girls, but in adolescents aged 16–18 years, it increased significantly. Log-binomial regression analysis showed that among all subjects, time (years), 16–18 years were negatively associated with thinness, while 13–15 years, walking to school, large family size, and paternal age at childbirth older than 30 years old were positively associated with thinness; 10–12/13–15/16–18 years, boarding at school, medium and large family sizes, and mother's education at junior middle school/junior high school and above were negatively associated with overweight/obesity, while time (years), boys were positively associated with overweight/obesity in the multivariate model by adjusting for the statistically significant factors (all *p* < 0.05).

**Conclusion:**

Chinese children and adolescents are facing a double burden of malnutrition. Future public health policies and interventions should prioritize high-risk groups specifically young age groups, boys, larger family sizes and so on.

## 1. Introduction

In recent decades, the United Nations has made the end of malnutrition the top priority of nutrition programs (1), but there has been little decrease in the prevalence of thinness and a significant increase in the prevalence of overweight/obesity among children and adolescents aged 5–19 years from 2010 to 2019, and few countries worldwide are projected to meet global nutrition goals by 2025 (2). In addition to malnutrition (protein energy malnutrition-thinness, vitamin A deficiency, anemia, iodine deficiency, and other micronutrient deficiencies), overnutrition (i.e., increased fat mass due to excessive energy intake, i.e., overweight and obesity and other noncommunicable diseases related to nutrition) also is an important global public health problem. They usually coexist in households and populations, with common underlying drivers and causes. This is the double burden of malnutrition (DBM) (3). The emergence of DBM is consistent with the trend of increasing globalization and changing dietary patterns. Although economic globalization may macroscopically accelerate economic development and increase national nutritional intake, it has also resulted in greater income and health inequities (4). These effects of globalization may slow progress in reducing undernutrition while increasing risk factors for overnutrition. Malnutrition in this study refers to thinness, and DBM has now been identified in many developing countries (5–9), including China, and it is critical to explore the current state of development and the drivers of DBM.

The growth and development of children and adolescents can be influenced by many factors at home, at school, and so on. During school age (usually 5 to 19 years), these factors could exacerbate or mitigate adversity in infancy and early childhood and, if developmentally healthy, would help to consolidate developmental outcomes in early childhood and correct some nutritional deficiencies and imbalances (10). Therefore, attention to the nutritional statuses of school-aged children and adolescents and the imposition of appropriate interventions are essential for their healthy transition to adulthood. Thinness was associated with premature mortality, physical suboptimal health, impaired neurocognitive function, and low productivity (9–11), and mortality and morbidity were 9–11 times higher in children with severe malnutrition than in well-nourished children (12); obesity in children and adolescents was associated with the development of ischemic heart disease (13), type II diabetes, respiratory disease, and psychological and social problems (14, 15) and had a strong tracking effect in adulthood (16). In addition, lifestyle changes such as less sleep time and physical activity and longer screen time in children and adolescents have been reported worldwide in recent years (17–20) and have played a negative role in the prevention and control of overweight and obesity.

China has experienced rapid economic development since the reform and opening up, and the nutritional status of children and adolescents has undergone a remarkable transformation from malnutrition to overweight and obesity (21). The 2014 National Students Physical Fitness and Health Survey showed that the overall prevalence of stunting in children and adolescents aged 7–18 years decreased from 16.4% in 1985 to 2.3% in 2014; the prevalence of thinness decreased from 8.4% to 4.0%; and the prevalence of overweight and obesity increased from 1.1% in 1985 to 20.4% in 2014 (21). It was expected that by 2030, the prevalence of overweight and obesity among school-age children aged 7 years and older would reach 28.0% and the number will reach 49.48 million (22). The Global Burden of Disease Study reported that the incidence of age-specific protein-energy malnutrition continued to decrease in China from 2010 to 2016. However, it soared to its highest point in 2017–2019 (23) and became more pronounced with lower age. The situation of malnutrition is still serious. Recent studies have shown that the prevalence of overweight and obesity in Chinese children and adolescents has continued to increase in recent years, but the rate has shown a downward trend (24, 25). According to different criteria for determining nutritional statuses, it was found that World Health Organization (WHO) criteria (26) and China criteria (27, 28) had similar determination rates for overweight and obesity, but China criteria had larger determination rates for thinness (29). In conclusion, DBM still exists in Chinese children and adolescents.

Sex and age (7, 8, 20) are factors associated with undernutrition and overnutrition, and sweet food intake of 1–3 times/week and 4–6 times/week are protective factors for thinness (30). The number of children in the family, the nutritional statuses of the parents, the conduct of physical education classes in schools, and the mode of travel were considered influential factors of overweight obesity (31, 32). There have also been a large number of studies on other behavioral factors such as diet, physical activity, sleep duration, sedentary time, socioeconomic status and the risk of thinness, overweight and obesity (17, 33–35). In general, recent studies on the factors influencing the nutritional statuses of Chinese children and adolescents have gathered more on diet and their behavioral patterns, and there is a slight lack of exploration on the factors of sociodemographic characteristics, and most of the studies have gathered on overweight and obesity, with insufficient studies on the factors influencing thinness.

Therefore, using a representative nationwide sample from 2010 to 2018, this study aimed to provide temporal trends in thinness, overweight, and obesity in Chinese children and adolescents, as well as to include different sociodemographic characteristic variables to explore their association with different nutritional statuses. For comparability at the national and international levels, this study used China and WHO body mass index (BMI) criteria to screen the nutritional statuses of children and adolescents.

## 2. Materials and methods

### 2.1. Study design and population

Data were from the Chinese Family Panel Studies (CFPS), a nationally representative longitudinal survey focusing on factors such as economic status, education level, and health status at the individual, household, and community levels, conducted by the Institute of Social Science Research at Peking University. The baseline assessment was conducted in 2010 using a multistage random sampling method and included 25 provinces, 16,000 family households and all household members in family households, representing 94.5% of the total Chinese population. An independent economic unit that resided in a traditional residential area and at least one person in the family had Chinese nationality could be considered a household that meets the project access conditions. Since 2010, the CFPS has had five follow-ups (2012, 2014, 2016, 2018, and 2020). All baseline household members and their future blood/adopted children, as defined by the 2010 baseline survey, will be permanently tracked as genetic members of the CFPS. Due to changes in household structure and the addition of new household members, new households and individuals are included in the following survey. In this paper, cross-sectional survey data for children and adolescents aged 7 to 18 years from the three CFPS surveys in 2010, 2014, and 2018 were selected, and participants who were interviewed at least once in 3 years of the survey were included, stratified by sex and age, with 1 year of age as 1 age group and 12 age groups for both sexes. The final sample included 12,234 participants, including 4,631 surveyed in 2010, 4,011 surveyed in 2014, and 3,592 surveyed in 2018, with a male-to-female ratio of approximately 1.1:1.

### 2.2. Procedures

All questionnaires were designed and standardized by Peking University and were available in both English and Chinese. Before the start of each survey, interviewers were recruited from the community. After successful registration, individuals were trained and assessed by Peking University, and a qualified interviewer certificate was issued. In 2010, the CFPS used face-to-face interviews. Since 2012, the CFPS has used a combination of telephone and face-to-face interviews, with rigorously trained interviewers conducting on-site visits or telephone interviews from the second half of the survey year until the first half of the following year. Information on children aged 15 and under was obtained from the pediatric database for each survey, mainly from their parents or primary guardians who answered on their behalf, and data on individuals aged 16 and older were obtained from the adult database (they answered the questions themselves). The variables for this study were measured as described below.

### 2.3. Anthropometric measurements and definitions

Body height and weight data were reported by their parents/guardians or by themselves based on individual self-reported or juvenile parent surrogate questionnaires for each survey year. The validity of self-reported height and weight in assessing childhood obesity was demonstrated with almost perfect agreement with measured values (36). Self-reported BMI values were highly correlated with measured BMI values (correlation coefficient 0.96) and both types of measurements were available, as reported in the Lancet (37). Therefore, self-reported body height and weight can be used in large-scale epidemiological studies when actual measurements are not possible. BMI = body weight (kg)/height^2^ (m^2^). Two age and sex-specific BMI criteria were used to define thinness, overweight, and obesity: (1) China criteria: According to the Screening for Overweight and Obesity in School-aged Children and Adolescents (WS/T 586-2018) (27) issued in 2018, BMI values greater than or equal to the overweight BMI cut-off values and less than the obesity BMI cut-off values by gender and age were judged as overweight, and BMI values greater than or equal to the obesity BMI cut-off values were determined as obese. In addition, individuals aged 18 years with 24 kg/m^2^ ≤ BMI < 28 kg/m^2^ were considered overweight and BMI ≥ 28 kg/m^2^ were considered obese. Screening Standard for Malnutrition of School-age Children and Adolescents (WS/T 456-2014) issued in 2014 (28) was used to discriminate malnutrition, which included moderate/severe thinness and mild thinness, combined as thinness, and BMI ≤ 18.5 kg/m^2^ for 18-year-old individuals were considered thinness; (2) WHO criteria: Gender-specific and age-specific BMI-Z scores were calculated according to WHO 2007 criteria (26). BMI-Z scores were divided into thinness (BMI Z score < −2), normal (−2 ≤ BMI Z score ≤ 1), overweight (1 < BMI Z score ≤ 2), and obese (BMI Z score > 2) groups. We excluded individuals with BMI Z scores >5 or < -5.

### 2.4. Sociodemographic determinants

The age of individuals surveyed in each year included the age in the survey year and the actual age (most of these surveys were completed in the current year, with a small number interviewed in the following year), and we selected the actual age for analysis; the 12 age groups of 7–18 years were divided into four age categories, 7–9 years (middle childhood in both sexes), 10–12 years (transition into puberty and midpuberty in most girls, transition into puberty in most boys), 13–15 years (later adolescence in most girls, interval of the growth spurt in most boys), and 16–18 years (later adolescence in almost all girls, later adolescence in most boys), based on the growth and development of children and adolescents; urban and rural were divided according to the criteria of the Chinese National Bureau of Statistics for all survey years; those who were not attending school and those who were attending kindergarten (< 5% of the total) were excluded because school factors were included in this study. For the school boarding variable, the question “Did your school provide boarding” was asked first, and the results included “Yes” or “No.” Then, we asked “Did you board at your school” for those individuals who answered “Yes,” and the results included “Yes” or “No.” Finally, the results of the two responses were combined. Individuals who did not board at school included both those who could not board at school and those who could board at school but did not board themselves; for travel to school (no data available for 16–18-year-olds in 2010), individuals were divided into those who walked to school (including walking by themselves or with a family member) and those who did not walk to school (including bicycles, motorcycles, buses, subways, school buses, private cars and so on) with consideration of physical activity. Although students who boarded in schools went home at different frequencies, they were all classified as non-walking in this study; for school types, they were classified as key/model schools and non-key schools; “Key class” meant that the school organized the students with higher scores from each class of a grade into one or two classes. They were instructed by excellent teachers. Usually, such a class was called “experimental class,” “dragon class,” or “tiger class.” Answers included yes, no, or the school attended did not distinguish between ordinary and key classes. Answers yes were classified as being in a key class, and the rest were non-key classes; family size included the number of family members related by marriage/blood/adoption, not necessarily including all immediate family members, and was divided into three sizes: small (≤3 individuals), medium (4–6 individuals), and large (≥7 individuals); parents' age at childbirth (referring to the parent's age at the time of birth), based on relevant literature (38), were divided into three groups: ≤25 years, 26–30 years, and >30 years, with ≤25 years as the control group; the highest completed education of parents represented their cultural education level. The answers included Illiterate/semiliterate, Primary school, Junior middle school, Junior high school, 3-Year College, 4-year college/Bachelor's degree, Master's degree, Doctoral degree (postdoctoral that was not mentioned in the questionnaire was included in doctoral degree by default) and Never been to study, which were divided into 3 levels, primary school and below, junior middle school, junior high school and above; family income (yuan/year) was the annual net income of all family members, which was more reflective of the annual economic level by deducting production costs from the total income. It was based on the self-reported annual income of all members living in the household, including income from all economic sources such as wages, funds, relief, and subsidies. It was recorded as 0 if the annual family income was negative. It is worth noting that the annual family income in 2010 refers to the past year, i.e., January 1, 2009, to December 31, 2009, while 2014 and 2018 refer to 12 months before the survey period. Subsequently, the annual family income for each survey year was divided equally into three categories (first to third quarters; the first quarter represented the lowest income and the third quarter represented the highest income) in descending order. Similarly, the annual per family income (yuan per/year) was obtained as annual family income/family size.

### 2.5. Statistical analysis

Individuals with any missing data were excluded based on the above-mentioned methods. The prevalence of thinness, overweight, and obesity by sex was calculated for each age group in each survey year according to the China and WHO BMI criteria. The chi-square test was performed to test the variability of sociodemographic variables in each group across years. Log-binomial regression was used to assess the statistical significance of temporal trends in the prevalence of thinness, overweight, and obesity throughout the entire period, using the year as a continuous variable by adjusting for age. The results of these analyses were expressed as prevalence ratios (PR) (95% CI). To facilitate comparison with most studies, data from 3 years were combined with WHO criteria using log-binomial regression to assess risk ratios between each group's sociodemographic characteristics and the prevalence of thinness, overweight/obesity (combining overweight and obesity). The univariate model controlled only for the target variable, and the multivariate model controlled for each variable that had a significant regression result in the univariate model. The target statuses (thinness, overweight/obesity) were coded as 1 and 0 otherwise, and the results were expressed as PR (*P*-value). We used logarithms as a link function to model the binary outcomes of log-binomial regression. All statistical tests were performed using a two-tailed test, and the level of statistical significance was set at 0.05. All analyses were conducted using IBM SPSS version 27.0 (IBM Corp, Armonk, NY, USA) and GraphPad Prism 9.3.1(GraphPad Software, Inc, CA, USA).

## 3. Results

Table 1 shows the basic information for this study. A total of 11,234 participants were included in the analysis for this study, with sample sizes of 4,631, 4,011, and 3,592 in 2010, 2014, and 2018, respectively. There were no significant differences between boys and girls, urban and rural areas, family size, family income (year), and family income per (year), but the numbers differed slightly by age group (*p* < 0.001). During the study period, more children and adolescents boarded at school, did not walk to school, were in key classes, and parents had higher ages at childbirth and higher education levels (all *p* < 0.001) (Table 1).

**Table 1:** Sociodemographic characteristics of children and adolescents aged 7–18 in the Chinese Family Panel Studies (CFPS) 2010, 2014, and 2018.

**Sociodemographic characteristics**	**CFPS 2010** ***N*** = **4,631**		**CFPS 2014** ***N*** = **4,011**		**CFPS 2018** ***N*** = **3,592**		P^*^
	***n***	**%**	***n***	**%**	***n***	**%**	
**Gender**							
Girls	2,259	48.8	1,954	48.7	1,676	46.7	0.108
Boys	2,372	51.2	2,057	51.3	1,916	53.3	
Age categories (years)							
7–9 years	1,077	23.3	1,075	26.8	649	18.1	< 0.001
10–12 years	1,413	30.5	1,160	28.9	1,135	31.6	
13–15 years	1,413	30.5	1,073	26.8	1,082	30.1	
16–18 years	728	15.7	703	17.5	726	20.2	
**Residential area**							
Rural	2,274	59.9	2,256	56.2	2,022	56.3	0.445
Urban	1,857	40.1	1,755	43.8	1,570	43.7	
**Boarding at school**							
No	3,305	71.4	2,833	70.6	2,379	66.2	< 0.001
Yes	1,326	28.6	1,178	29.4	1,213	33.8	
**Walking to school**							
No	1,432	30.9	2,402	59.9	2,404	66.9	< 0.001
Yes	2,471	53.4	1,609	40.1	1,188	33.1	
No data	728	15.7	0	0.0	0	0.0	
**Key/Model School**							
No	4,343	93.8	2,987	74.5	2,641	73.5	< 0.001
Yes	288	6.2	1,024	25.5	951	26.5	
**Key class**							
No	4,171	90.1	3,554	88.6	3,139	87.4	< 0.001
Yes	460	9.9	457	11.4	453	12.6	
**Family size**							
Small	915	19.8	767	19.1	669	18.6	0.064
Medium	3,152	68.1	2,704	67.4	2,409	67.1	
Large	564	12.2	540	13.5	514	14.3	
**Paternal age at childbirth (year)**							
≤ 25	1,702	36.8	1,308	32.6	1,187	33.0	< 0.001
26~	1,856	40.1	1,534	38.2	1,257	35.0	
>30	1,073	23.2	1,169	29.1	1,148	32.0	
**Maternal age at childbirth (year)**							
≤ 25	2,450	52.9	1,925	48.0	1,789	49.8	< 0.001
26~	1,518	32.8	1,309	32.6	1,042	29.0	
>30	663	14.3	777	19.4	761	21.2	
**Father's education**							
Primary school and below	1,962	42.4	1,701	42.4	1,280	35.6	< 0.001
Junior middle school	1,769	38.2	1,458	36.4	1,402	39.0	
Junior high school and more	900	19.4	852	21.2	910	25.3	
**Mather's education**							
Primary school and below	2,638	57.0	2,147	53.5	1,583	44.1	< 0.001
Junior middle school	1,336	28.8	1,248	31.1	1,287	35.8	
Junior high school and more	657	14.2	616	15.4	722	20.1	
**Family income (year)**							
Quintile 1 (lowest quintile)	1,564	33.8	1,324	33.0	1,179	32.8	0.577
Quintile 2	1,595	34.4	1,350	33.7	1,224	34.1	
Quintile 3 (highest quintile)	1,472	31.8	1,337	33.3	1,189	33.1	
**Family income per (year)**							
Quintile 1 (lowest quintile)	1,470	31.7	1,319	32.9	1,205	33.5	0.464
Quintile 2	1,564	33.8	1,353	33.7	1,198	33.4	
Quintile 3 (highest quintile)	1,597	34.5	1,339	33.4	1,189	33.1	

According to the number of individuals living in the family, family size was classified as small (≤ 3), medium (4–6) and large (≥7).

Family income (Yuan/year): CFPS 2010: Q1 (≤ 15,000), Q2 (15000.1–30000.0), Q3 (> 30000.0); CFPS 2014: Q1 (≤ 27000), Q2 (27000.1–55000.0), Q3 (> 55000.0); CFPS 2018: Q1 (≤ 45,000), Q2 (45000.1–86000.0), Q3 (> 86000.0).

Family income per (Yuan/year): CFPS 2010: Q1 (≤ 3,000), Q2 (3000.1–6500.0), Q3 (> 6500.0); CFPS 2014: Q1 (≤ 5,500), Q2 (5500.1–12000.0), Q3 (> 12000.0); CFPS 2018: Q1 (≤ 9,500), Q2 (9500.1–18500.0), Q3 (> 18500.0).

* The p-values were obtained from the chi-square tests for categorical variables. Same below.

**Figure 1:**
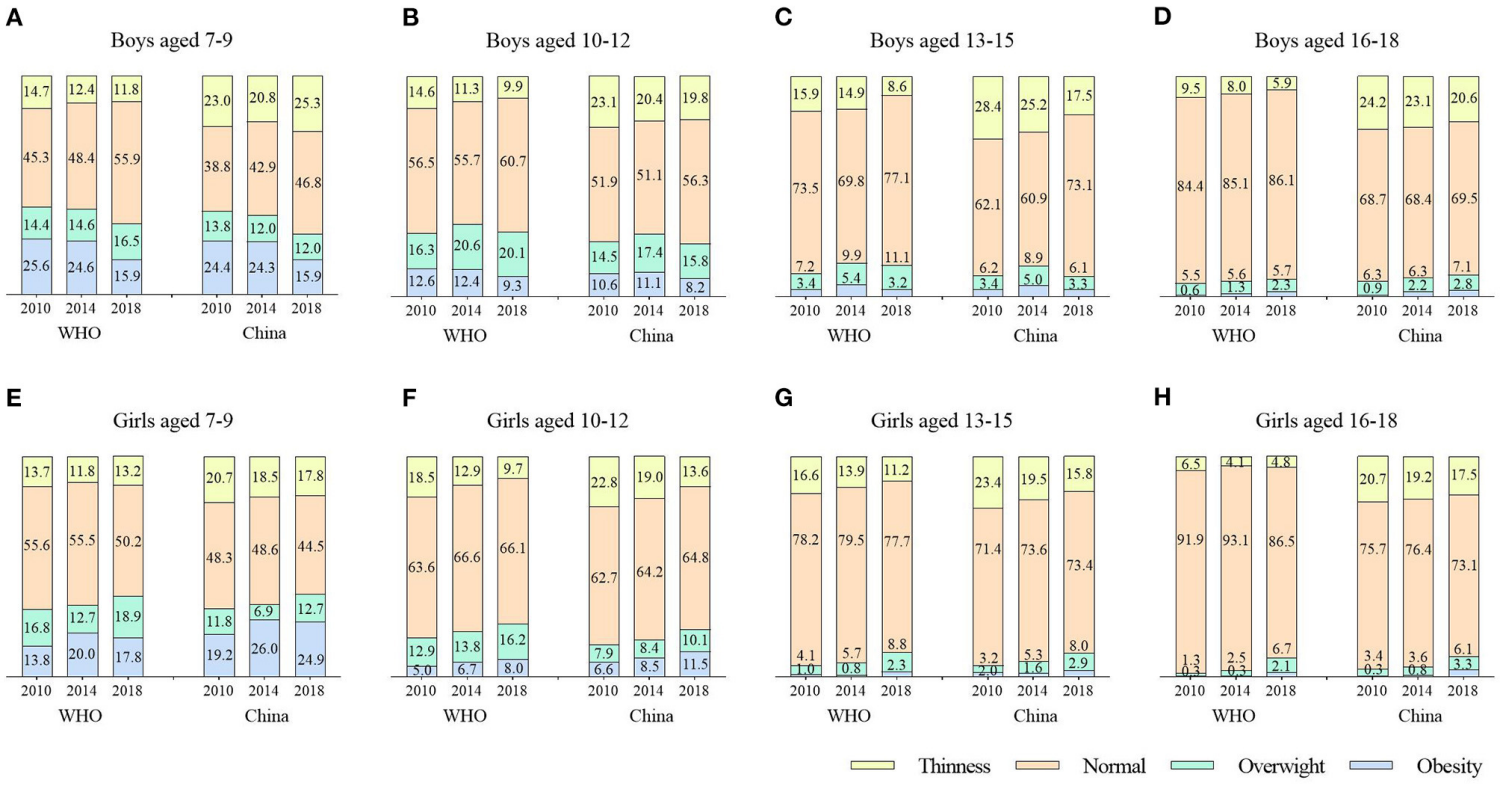
Distribution of nutritional statuses based on BMI and defined by the China and WHO criteria according to gender and age [7–9 years boys **(A)** and girls **(E)**, 10–12 years boys **(B)** and girls **(F)**, 13–15 years boys **(C)** and girls **(G)**, 16–18 years boys **(D)** and girls **(H)**] in the Chinese Family Panel Studies (CFPS) 2010, 2014 and 2018, China.

**Figure 2:**
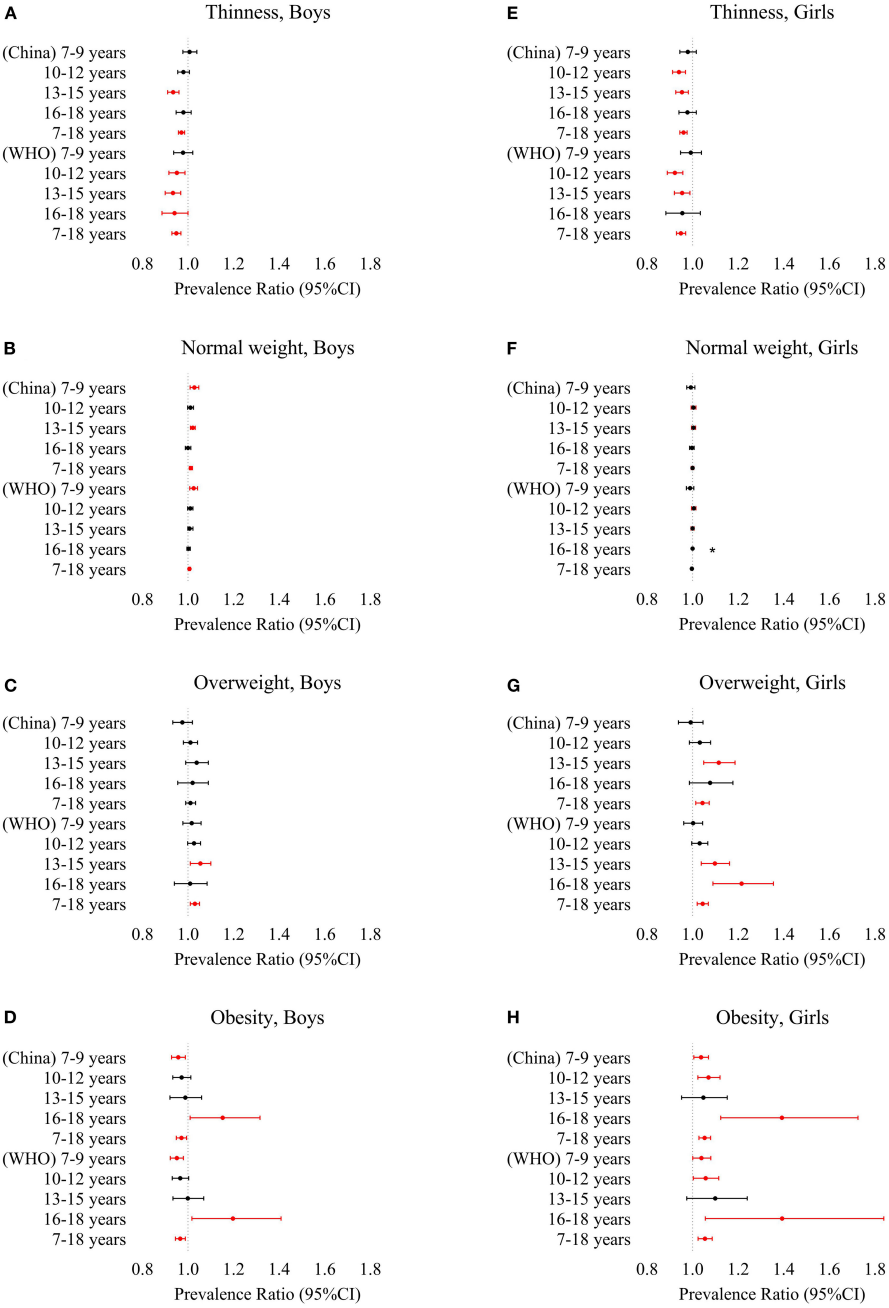
Prevalence ratio by log-binomial regression with year as a continuous variable and adjusted for age of thinness, overweight and obesity based on the China and WHO criteria according to gender and age (7–9 years, 10–12 years, 13–15 years, 16–18 years, 7–18 years) from 2010 to 2018 [thinness boys **(A)** and girls **(E)**, normal weight boys **(B)** and girls **(F)**, overweight boys **(C)** and girls **(G)**, obesity boys **(D)** and girls **(H)**]. Variables that were significantly associated with thinness risk are shown in red (*p* < 0.05). *The log-binomial regression model cannot be fitted.

Figure 1 shows the prevalence of thinness, overweight, and obesity. Figure 2 shows the log-binomial regression analysis of thinness and overweight/obesity. After adjusting age, the prevalence of thinness in boys decreased from 13.7% in 2010 to 9.1% in 2018 according to WHO criteria; from 24.7% in 2010 to 20.8% in 2018 according to China criteria. The prevalence of thinness in girls decreased from 13.8% in 2010 to 9.7% in 2018 according to WHO criteria; from 21.9% in 2010 to 16.2% in 2018 according to China criteria. The prevalence rate was higher according to the China criteria. Over time, the prevalence of thinness in all age groups of both sexes (WHO criteria: boys, PR = 0.949, 95% CI, 0.929–0.969; girls, PR = 0.949.95% CI, 0.929–0.970; Chinese criteria: boys, PR = 0.971, 95% CI, 0.958–0.985; girls, PR = 0.960, 95% CI, 0.944–0.976) significantly decreased, all of which were statistically significant. By age categories, there were no significant trends in the prevalence of thinness in children aged 7–9 years and adolescents aged 16–18 years (except for boys as defined by the WHO). Overall, the prevalence of thinness among Chinese children and adolescents aged 7–18 years decreased during the period 2010–2018.

The age-adjusted prevalence of normal weight in boys increased from 64.9% in 2010 to 70.0% in 2018 according to WHO criteria; from 55.4% in 2010 to 61.3% in 2018 according to China criteria. The prevalence of normal weight in girls declined from 72.3% in 2010 to 70.1% in 2018 according to WHO criteria; from 64.5% in 2010 to 63.9% in 2018 according to China criteria. The rate of China criteria was low. Over time, only the prevalence of normal weight in boys (WHO criteria: PR = 1.006, 95% CI, 1.002–1.011; China criteria: PR = 1.012, 95% CI, 1.006–1.019) increased significantly, all of which were statistically significant. By age categories, significant increases were observed in boys aged 7–9 (WHO and China criteria) and 13–15 years (China criteria). Overall, the prevalence of overweight among Chinese children and adolescents aged 7–18 years increased during the period 2010–2018 in boys, with a stable trend in girls.

The age-adjusted prevalence of overweight in boys increased from 10.9% in 2010 to 14.4% in 2018 according to WHO criteria; from 10.2% in 2010 to 10.3% in 2018 according to China criteria. The prevalence of overweight in girls increased from 8.8% in 2010 to 12.7% in 2018 according to WHO criteria; from 6.6% in 2010 to 9.2% in 2018 according to China criteria. The rate of China criteria was low. Over time, except for the boys of China criteria, the prevalence of overweight in boys and girls (WHO criteria: boys, PR = 1.030, 95% CI, 1.010–1.050; girls, PR = 1.044, 95% CI, 1.020–1.069; China criteria: girls, PR = 1.043, 95% CI, 1.014–1.073) increased significantly, all of which were statistically significant. By age categories, the prevalence of overweight was higher for boys and girls in the lower age categories. Significant increases were observed at 13–15 years of age except for boys of China criteria, in addition to girls aged 16–18 years of WHO criteria (all *p* < 0.05). Overall, the prevalence of overweight among Chinese children and adolescents aged 7–18 years increased during the period 2010–2018, with a faster increase among girls than boys.

The age-adjusted prevalence of obesity in boys decreased from 10.6% in 2010 to 7.7% in 2018 according to WHO criteria; from 9.8% in 2010 to 7.6% in 2018 according to China criteria. The prevalence of obesity in girls increased from 5.0% in 2010 to 7.6% in 2018, according to WHO criteria; from 7.0% in 2010 to 10.7% in 2018, according to Chinese criteria. The prevalence was higher for the China criteria. Over time, the prevalence of obesity among boys and girls showed an opposite trend, with a decrease in boys (WHO criteria: PR = 0.966, 95% CI, 0.945–0.988; China criteria: PR = 0.971, 95% CI, 0.949–0.994) and an increase in girls (WHO criteria: PR = 1.054, 95% CI, 1.024–1.086; China criteria: PR = 1.053, 95% CI, 1.028–1.079), all of which were statistically significant. By age categories, significant increases were observed in girls except for 13–15 years (all *p* < 0.05); in boys, although the overall prevalence decreased, it increased in boys aged 16–18 years (*p* < 0.05). Overall, the prevalence of obesity decreased among boys and increased among girls aged 7–18 years in China during the period 2010–2018, with a trend exceeding that of boys (Figures 1, 2).

Table 2 shows the log-binomial regression analysis of thinness and sociodemographic variables. In these univariate models, across all subjects, time (years), 16–18 years, urban residential area, boarding at school, being enrolled in a key/model school, being enrolled in a key class, father's education at junior high school and above, mother's education at junior middle school/junior high school and above, and family income per at Quintile 3 were independently negatively associated with thinness, while walking to school, medium and large family sizes, paternal age at childbirth older than 30 years old were independently positively associated with thinness (all *p* < 0.05). In the multivariate model, by adjusting for the above statistically significant factors, we found that time (years), 16–18 years were negatively associated with thinness (PR = 0.958, 95% CI 0.943–0.974; PR = 0.589, 95% CI 0.460–0.755, respectively), while 13–15 years, walking to school, large family size, and paternal age at childbirth older than 30 years old were positively associated with thinness (all *p* < 0.05) (PR = 1.146, 95% CI 1.003–1.308; PR = 1.171, 95% CI 1.046–1.310; PR = 1.250, 95% CI 1.041–1.501; PR = 1.153, 95% CI 1.016–1.307, respectively) (Table 2).

**Table 2:** Log-binomial regression analysis between thinness and sociodemographic variables in Chinese children and adolescents aged 7–18 years in the Chinese Family Panel Studies (CFPS) 2010, 2014, and 2018.

**Sociodemographic characteristics**	***N***	***n* (%)**	**Univariate model**		**Multivariate model**	
			***PR*** **(95%** ***CI*****)**	***P***	***PR*** **(95%** ***CI*****)**	***P***
Year	12,234	1,484 (12.1)	0.947 (0.933, 0.962)	**<** **0.001**	0.958 (0.943, 0.974)	**<** **0.001**
**Age categories (years)**						
7–9 years	2,801	364 (13.0)	1	**—**	1	
10–12 years	3,708	483 (13.0)	1.002 (0.883, 1.138)	0.971	1.019 (0.897, 1.157)	0.773
13–15 years	3,568	492 (13.8)	1.061 (0.935, 1.204)	0.357	1.146 (1.003, 1.308)	**<** **0.05**
16–18 years	2,157	145 (6.7)	0.517 (0.430, 0.622)	**<** **0.001**	0.589 (0.460, 0.755)	**<** **0.001**
**Gender**						
Girls	5,889	724 (12.3)	1	—	—	—
Boys	6,345	760 (12.0)	0.974 (0.886, 1.072)	0.592	—	—
**Residential area**						
Rural	7,052	917 (13.0)	1	—	1	—
Urban	5,182	567 (10.9)	0.841 (0.763, 0.928)	**<** **0.001**	0.920 (0.822, 1.030)	0.148
**Boarding at school**						
No	8,517	1,122 (13.2)	1	—	1	—
Yes	3,717	362 (9.7)	0.739 (0.661, 0.827)	**<** **0.001**	0.899 (0.782, 1.034)	0.135
**Walking to school**						
No	6,238	650 (10.4)	1	—	1	—
Yes	5,268	772 (14.7)	1.406 (1.276, 1.551)	**<** **0.001**	1.171 (1.046, 1.310)	**<** **0.01**
No data	728	—				
**Key/Model School**						
No	9,971	1,262 (12.7)	1	—	1	—
Yes	2,263	222 (9.8)	0.775 (0.677, 0.887)	**<** **0.001**	0.984 (0.851, 1.138)	0.830
**Key class**						
No	10,864	1,353 (12.5)	1	—	1	—
Yes	1,370	131 (9.6)	0.768 (0.648, 0.910)	**<** **0.01**	0.948 (0.792, 1.135)	0.564
**Family size**						
Small	2,351	239 (10.2)	1	—	1	—
Medium	8,265	1,013 (12.3)	1.206 (1.055, 1.378)	**<** **0.01**	1.108 (0.961, 1.278)	0.158
Large	1,618	232 (14.3)	1.410 (1.191, 1.670)	**<** **0.001**	1.250 (1.041, 1.501)	**<** **0.05**
**Paternal age at childbirth (year)**						
≤ 25	4,197	479 (11.4)	1	—	1	—
26~	4,647	567 (12.2)	1.069 (0.954, 1.098)	0.252	1.071 (0.952, 1.204)	0.255
>30	3,390	438 (12.9)	1.131 (1.003, 1.278)	**<** **0.05**	1.153 (1.016, 1.307)	**<** **0.05**
**Maternal age at childbirth (year)**						
≤ 25	6,164	752 (12.2)	1	—	—	—
26~	3,869	461 (11.9)	0.977 (0.876, 1.089)	0.670	—	—
>30	2,201	271 (12.3)	1.009 (0.886, 1.149)	0.890	—	—
**Father's education**						
Primary school and below	4,943	649 (13.1)	1	—	1	—
Junior middle school	4,629	571 (12.3)	0.939 (0.846, 1.044)	0.244	1.023 (0.914, 1.146)	0.691
Junior high school and more	2,662	264 (9.9)	0.755 (0.660, 0.865)	**<** **0.001**	0.896 (0.759, 1.058)	0.196
**Mather's education**						
Primary school and below	6,368	853 (13.4)	1	—	1	—
Junior middle school	3,871	430 (11.1)	0.829 (0.744, 0.925)	**<** **0.001**	0.916 (0.812, 1.033)	0.151
Junior high school and more	1,995	201 (10.1)	0.752 (0.650, 0.870)	**<** **0.001**	0.932 (0.776, 1.119)	0.452
**Family income (year)**						
Quintile 1 (lowest quintile)	4,067	521 (12.8)	1	—	—	—
Quintile 2	4,169	498 (11.9)	0.932 (0.831, 1.046)	0.233	—	—
Quintile 3 (highest quintile)	3,998	465 (11.6)	0.908 (0.808, 1.021)	0.106	—	—
**Family income per (year)**						
Quintile 1 (lowest quintile)	3,994	515 (12.9)	1	—	1	—
Quintile 2	4,115	517 (12.6)	0.974 (0.869, 1.092)	0.655	1.065 (0.947, 1.197)	0.294
Quintile 3 (highest quintile)	4,125	452 (11.0)	0.850 (0.755, 0.957)	**<** **0.01**	1.000 (0.875, 1.143)	0.999

PR, prevalence ratios; CI, confidence interval; Univariate model controls only for the target variable, and the multivariate model controls for each variable that has a significant regression result in the univariate model.

The bold values represented significance.

Binomial logistic regression analysis of the relationship between sociodemographic characteristics and overweight/obesity is shown in Table 3. In these univariate models, 10–12/13–15/16–18 years, boarding at school, being enrolled in a key class, and medium family size were independently negatively associated with overweight/obesity, while time (years), boys, walking to school, mother's education at junior high school and above, and family income at Quintile 3 were independently positively associated with overweight/obesity (all *p* < 0.05). In the multivariate model, by adjusting for the above statistically significant factors, we found that 10–12/13–15/16–18 years, boarding at school, medium and large family sizes, mother's education at junior middle school/junior high school and above were negatively associated with overweight/obesity (PR = 0.742, 95% CI 0.689–0.799; PR = 0.303, 95% CI 0.271–0.340; PR = 0.198, 95% CI 0.161–0.244; PR = 0.805, 95% CI 0.719–0.900; PR = 0.850, 95% CI 0.780–0.927; PR = 0.852, 95% CI 0.755–0.961; PR = 0.922, 95% CI 0.853–0.997; PR = 0.847, 95% CI 0.763–0.940, respectively), while time (years), and boys were positively associated with overweight/obesity (PR = 1.016, 95% CI 1.005–1.027; PR = 1.337, 95% CI 1.247–1.434, respectively) (all *p* < 0.05) (Table 3).

**Table 3:** Log-binomial regression analysis between overweight/obesity and sociodemographic variables in Chinese children and adolescents aged 7–18 years in the Chinese Family Panel Studies (CFPS) 2010, 2014, and 2018.

**Sociodemographic characteristics**	***N***	***n* (%)**	**Univariate model**		**Multivariate model**	
			***PR*** **(95%** ***CI*****)**	***P***	***PR*** **(95%** ***CI*****)**	***P***
Year	12,234	2,442 (12.1)	1.012 (1.002, 1.023)	**<** **0.05**	1.016 (1.005, 1.027)	**<** **0.01**
**Age categories (years)**						
7–9 years	2,801	987 (35.2)	1	**—**	1	**—**
10–12 years	3,708	962 (25.9)	0.736 (0.684, 0.793)	**<** **0.001**	0.742 (0.689, 0.799)	**<** **0.001**
13–15 years	3,568	366 (10.3)	0.291 (0.261, 0.325)	**<** **0.001**	0.303 (0.271, 0.340)	**<** **0.001**
16–18 years	2,157	127 (5.9)	0.167 (0.140, 0.199)	**<** **0.001**	0.198 (0.161, 0.244)	**<** **0.001**
**Gender**						
Girls	5,889	970 (16.5)	1	—	1	—
Boys	6,345	1,472 (23.2)	1.408 (1.309, 1.515)	**<** **0.001**	1.337 (1.247, 1.434)	**<** **0.001**
**Residential area**						
Rural	7,052	1,417 (20.1)	1	—	—	—
Urban	5,182	1,025 (19.8)	0.984 (0.916, 1.058)	0.668	—	—
**Boarding at school**						
No	8,517	2,048 (24.0)	1	—	1	—
Yes	3,717	394 (10.60)	0.441 (0.399, 0.488)	**<** **0.001**	0.805 (0.719, 0.900)	**<** **0.001**
**Walking to school**						
No	6,238	1,192 (19.1)	1	—	1	—
Yes	5,268	1,220 (23.2)	1.212 (1.129, 1.301)	**<** **0.001**	0.941 (0.874, 1.013)	0.105
No data	728	—				
**Key/Model School**						
No	9,971	2,011 (20.2)	1	—	—	—
Yes	2,263	431 (19.0)	0.944 (0.860, 1.037)	0.230	—	—
**Key class**						
No	10,864	2,241 (20.6)	1	—	1	—
Yes	1,370	201 (14.7)	0.711 (0.623, 0.812)	**<** **0.001**	1.089 (0.959, 1.237)	0.189
**Family size**						
Small	2,351	505 (21.5)	1	—	1	—
Medium	8,265	1,598 (19.3)	0.900 (0.824, 0.984)	**<** **0.05**	0.850 (0.780, 0.927)	**<** **0.001**
Large	1,618	339 (21.0)	0.975 (0.863, 1.102)	0.689	0.852 (0.755, 0.961)	**<** **0.01**
**Paternal age at childbirth (year)**						
≤ 25	4,197	837 (19.9)	1	—	—	—
26~	4,647	880 (18.9)	0.950 (0.872, 1.034)	0.232	—	—
>30	3,390	735 (21.4)	1.072 (0.982, 1.172)	0.122	—	—
**Maternal age at childbirth (year)**						
≤ 25	6,164	1,236 (20.1)	1	—	—	—
26~	3,869	737 (19.0)	0.950 (0.875, 1.031)	0.219	—	—
>30	2,201	469 (21.3)	1.063 (0.967, 1.168)	0.207	—	—
**Father's education**						
Primary school and below	4,943	963 (19.5)	1	—	—	—
Junior middle school	4,629	923 (19.9)	1.023 (0.944, 1.110)	0.574	—	—
Junior high school and more	2,662	556 (20.9)	1.072 (0.977, 1.177)	0.143	—	—
**Mather's education**						
Primary school and below	6,368	1,214 (19.1)	1	—	1	—
Junior middle school	3,871	798 (20.6)	1.081 (0.998, 1.171)	0.055	0.922 (0.853, 0.997)	**<** **0.05**
Junior high school and more	1,995	430 (21.6)	1.131 (1.025, 1.247)	**<** **0.05**	0.847 (0.763, 0.940)	**<** **0.01**
**Family income (year)**						
Quintile 1 (lowest quintile)	4,067	767 (18.9)	1	—	1	—
Quintile 2	4,169	818 (19.6)	1.040 (0.952, 1.137)	0.381	1.024 (0.955, 1.141)	0.589
Quintile 3 (highest quintile)	3,998	857 (21.4)	1.137 (1.042, 1.240)	**<** **0.05**	1.044 (0.940, 1.114)	0.344
**Family income per (year)**						
Quintile 1 (lowest quintile)	3,994	805 (20.2)	1	—	—	—
Quintile 2	4,115	772 (18.8)	0.931 (0.852, 1.017)	0.113	—	—
Quintile 3 (highest quintile)	4,125	865 (21.0)	1.040 (0.955, 1.133)	0.364	—	—

PR, prevalence ratios; CI, confidence interval; Univariate model controls only for the target variable, and the multivariate model controls for each variable that has a significant regression result in the univariate model.

The bold values represented significance.

## 4. Discussion

This was a study describing both secular trends and sociodemographic determinants of thinness and overweight and obesity in Chinese children and adolescents. This study showed that the prevalence of thinness among Chinese children and adolescents aged 7–18 years decreased during the entire period, mainly in the 10–15 years age categories, with the same trend for both sexes. The prevalence of thinness was slightly greater among girls than boys. The prevalence of overweight increased for both boys and girls, and the prevalence was greater for boys than girls, but the prevalence trends of obesity were reversed for both sexes, with boys decreasing and girls increasing to the level of boys. We also found significant increases in the prevalence of obesity for boys and girls aged 16–18 years.

One study showed that the prevalence of malnutrition among Chinese boys and girls decreased from 8.0–9.0% in 2015 to 3.0–4.0% in 2021 using the China criteria (39), which was consistent with our study but with significantly lower prevalence. It was worth noting that this study did not classify mild thinness as thinness, whereas some studies in China were included. If mild thinness is not classified as thinness, the prevalence of thinness using China criteria and WHO criteria would be closer. Further research and exploration by national health and sanitation departments are needed to determine the classification criteria. According to the report of the Global Burden of Disease (GBD) in 2019, the average annual percentage change (AAPC) of the age-specific incidence of protein-energy malnutrition from 1990 to 2019 was positive in China for ages 5–19 years (5–9, 10–14, and 15–19 years), but the annual percentage change (APC) was negative in 2010–2017, indicating that this period of protein-energy malnutrition was under control. Surprisingly, the APC increased significantly to approximately three times the previous fastest period in 2017–2019 (40). A possible reason for this phenomenon is that the determination used for GDB is based on body mass-height z-score (WHZ). A study of children and adolescents aged 7–17 years from 2000 to 2018 showed that overall prevalence of overweight and obesity continued to increase for both boys and girls, but the rate slowed down from 2015 to 2018 and even a downward trend was observed among urban boys (25). Yuan et al. (24) found in a study of children aged 6–15 years from 2009/2010 to 2017/2019 that 6–9-year-old boys and 6–10-year-old girls had a decreased prevalence of obesity and an increased prevalence in the remaining age groups. Combined with our study, in contrast to the previous sustained rise in all subgroups (21), obesity among Chinese children and adolescents has shown a positive trend in recent years.

Overall the prevalence of thinness was consistent in boys and girls, and the prevalence of overweight and obesity was higher in boys, consistent with previous studies (7, 8, 20, 24, 25). However, some studies showed that girls were more likely to be stunted or thin (6, 21) and others demonstrated that boys were more likely to be stunted or thin (29, 39). It was speculated that these differences may be related to the country and the different regions. In addition, we found that although the overall prevalence of obesity in boys had decreased, it had increased substantially for boys aged 16–18 years as well as for girls. In recent years, China has strengthened intervention measures for overweight and obesity in childhood, parents' nutritional concepts have gradually improved, schools and society have paid increasing attention to children, and prevention and control have been effective, causing the prevalence to slow down or decrease. However, for junior high school students approaching adulthood, schools and parents had given students greater autonomy to make choices, resulting in conditions and opportunities for students to adopt unhealthy diets and lifestyles. Moreover, Chinese students in junior high school were under increasing pressure to pass National College Entrance Examination (NEMT), creating negative behavioral patterns such as less physical activity (41) and increasing the incentives to develop obesity. However, it might also be related to the low number of obese individuals in this age cohort, where a very small increase in the number of individuals can cause a significant increase in prevalence.

Regression analysis showed that the prevalence of thinness was lower for urban children and adolescents, but the prevalence of overweight and obesity had significant urban-rural characteristics. There was no significant difference in the prevalence in the multifactorial model. Previous studies had shown that three decades ago, the prevalence of thinness in Chinese rural children and adolescents was significantly higher than that in urban areas, and the prevalence of overweight and obesity was significantly lower than that in urban areas; over time, the prevalence of thinness in rural areas decreased significantly, and the prevalence of overweight and obesity was almost close to that in urban areas. It was also found that the prevalence of overweight and obesity in rural areas was increasing faster than that in urban areas (21, 42). Hu et al. (25) also found that the increase in BMI of children and adolescents in rural areas was greater than that in urban areas. This further suggests that China should pay attention to the dissemination of nutritional concepts and balanced development of each region while actively building the countryside and ensuring nutritional needs. The older the age, the lower the prevalence of thinness and overweight and obesity in all subgroups, consistent with related studies (6, 8, 20). Some studies also showed opposite results or little difference among different age categories (18).

Regarding school factors, boarding at school, being enrolled in a key/model school, and being enrolled in a key class were protective factors for thinness (PR < 1); walking to school was a risk factor. Boarding at school and being enrolled in a key class were protective factors for overweight/obesity; walking to school was a risk factor. After adjusting for other factors, only walking to school was a risk factor for thinness and boarding at school was a protective factor for overweight/obesity. We suggest that with the introduction of school nutrition programs in recent years, schools could provide nutritious diets and monitor students' intake of extra snacks. Without boarding, students would be more likely to develop DBM if their diet was not nutritionally complete due to parental permissiveness (31). Ma et al. (43) found that eating out three or more times per week was associated with a higher prevalence of overweight/obesity in boys and not in girls. Students boarding at school effectively inhibited the frequency of eating out, specifically for boys. Walking to school was a risk factor for thinness. Zhu et al. (32) also noted that dynamic commuting was not correlated with overweight/obesity. A meta-study showed that older, boys, and underweight adolescents were more likely to use dynamic commuting to and from school (44), and the causal relationship was thought to be that dynamic commuting increases physical activity, and increased physical activity was associated with a lower prevalence of overweight/obesity (20, 45). Whether or not to walk to school might also be related to factors such as family income and parental jobs, and further research would be needed to control for confounding factors.

For family factors, the larger the family size, the greater the likelihood of being thin and the less likely to being overweight and obese for children and adolescents. Larger family size meant more children, a more dispersed distribution of food, and a greater likelihood of being thinner compared to devoting all resources to one individual. Some studies have found only a child to be a risk factor for obesity, somewhat similar to the present study (31, 46, 47). The prevalence of thinness was found to be higher in boys only when the paternal age at childbirth was older than 30 years old. One study pointed out that paternal age at childbirth can cause earlier pubertal development in both sexes (38), but there are few studies about malnutrition. Savage et al. (48) found a decrease in BMI in children with increasing maternal age at childbirth. However, other studies reported no impact of maternal age on BMI of children (49, 50). Recent data indicated that children of fathers aged >35 years at childbirth had lower BMI than children of fathers aged 31–35 and ≤30 years (51). In China, a higher paternal age may have negative effects on children's health; that is, higher income families can afford more high-energy foods and access more sedentary modes of transportation and leisure (52). In summary, the impact of paternal and maternal age at childbirth on nutritional statuses is currently unclear. Given the secular trend of older parents and the recent implementation of the Chinese two-child policy, further investigation is needed to verify our findings.

We found more similar effects of education level and family income variables on the nutritional statuses of children and adolescents. Higher indicators both corresponded to lower prevalence of thinness and higher prevalence of overweight/obesity. However, after adjusting for other factors, we only found that higher maternal education was associated with a lower prevalence of overweight/obesity in their offspring. Parents with higher levels of education might have higher nutritional knowledge and economic statuses, and there were some associations between the two sets of variables. Some studies using years of education to represent education level and using high tuition schools to represent the socioeconomic statuses of the family found similar results to the present study (5–7, 14, 32). It appeared that the mother's education was more associated with overweight/obesity compared to the father's (32, 45). Zegleń et al. generalized the high socioeconomic status to a small parental birthplace population, a high child number, and high education level as a combined variable and found lower thinness and higher overweight/obesity for high socioeconomic status (53). Some studies also noted that the prevalence of obesity was lower in girls with higher annual family income (31) and that children with higher father's education were less likely to be overweight/obese (46), which was inconsistent with the present study.

The first strength of our study was that we were able to design it using three nationally representative, large-scale population datasets, a sample that covers most of the provinces in China and reflects almost the entire country in general. In addition, the data were collected by highly trained staff using standardized interviewing instruments, and the data were judged and organized for reasonableness. Different criteria were used to determine the nutritional statuses based on BMI cut-off values for comparison. For the selection of sociodemographic characteristics variables, we included three aspects: individual characteristics, school characteristics, and family characteristics for a more comprehensive interpretation of the results. Compared with traditional binary logistic regression, binomial log regression could avoid overestimation of the risk ratio when the target rate was high, and this was one of the few studies using this regression analysis with a large sample in China. However, there were some limitations in this study. First, the body shape information was self-reported, and although its usability in large sample studies had been confirmed, this could still lead to some errors. Second, this was a cross-sectional study. Although some factors were found to be significantly associated with the nutritional status of children and adolescents, a causal relationship could not be verified. Third, we did not explore the effects of physical activity, school physical education, family activities, and diet on malnutrition in children and adolescents. Finally, despite the large sample sizes of the three CFPS surveys, some stratified analyses might be inadequate, no separate regressions were conducted for each year's variables, and it was unclear whether time plays a role in the effector factors.

## 5. Conclusions

In conclusion, Chinese children and adolescents had a dual burden of malnutrition, especially children. The prevalence of thinness decreased among boys and girls, but the prevalence of overweight and obesity increased, especially among girls and older adolescents. The double burden of malnutrition in China was gender and age-specific and was of overall concern and should be taken into account. The analysis of sociodemographic characteristics also informed the development of future public health policies and interventions. For example, emphasis needed to be placed on comprehensive education and dissemination of knowledge about healthy nutrition, raising parents' awareness of the impact of their children's weight, especially among schoolchildren. Using different strategies to promote a balanced diet and nutritional quality and motivating children to engage in a wider variety of physical activities to maintain a weight appropriate for age and height. In the school environment, rigorous implementation of physical activity programs should be ensured and students should be given sufficient time for after-school activities. School-applied cafeteria meals should consider a balanced ratio of various nutrients and micronutrients to encourage children and adolescents to eat healthily and develop good behaviors.

## Data availability statement

Publicly available datasets were analyzed in this study. This data can be found at: http://www.isss.pku.edu.cn/cfps/xgxw/cfpsdt/index.htm. Chinese Family Panel Studies.

## Ethics statement

The studies involving human participants were reviewed and approved by Medical Research Ethics Committee of the Peking University Health Science Center (IRB00001052-14010), Peking University. Written informed consent to participate in this study was provided by the participants' legal guardian/next of kin.

## Author contributions

CL, MZ, AT, YC, QL, and HW contributed to the conception and design of the study. MZ and QL organized the database. CL, YC, and HW performed the statistical analysis. CL wrote the first draft of the manuscript. MZ, AT, YC, QL, and HW wrote sections of the manuscript. CL and HW used software to make figures. AT and YC reviewed and edited the article. All authors contributed to manuscript revision, read, and approved the submitted version.
